# Management of a Large Post-tonsillectomy Thrombus Obstructing the Laryngeal View: A Case Report

**DOI:** 10.7759/cureus.46763

**Published:** 2023-10-09

**Authors:** Rana Tamraz, Roman Austin, Ricardo Falcon, Tania Kraai, Richard Lock, Timothy R Petersen, Codruta Soneru

**Affiliations:** 1 Anesthesiology and Critical Care Medicine, University of New Mexico School of Medicine, Albuquerque, USA; 2 Otolaryngology - Head and Neck Surgery, University of New Mexico School of Medicine, Albuquerque, USA; 3 Office of Graduate Medical Education, University of New Mexico School of Medicine, Albuquerque, USA

**Keywords:** obstructed laryngoscopic view, difficult intubation, post-tonsillectomy complication, post-tonsillectomy hemorrhage, post-tonsillectomy bleed, blood clot, airway

## Abstract

Anesthetic management of children with a post-tonsillectomy hemorrhage can be challenging. The patients may be anemic and hypovolemic and are at increased risk of having a difficult airway due to active bleeding, vomiting, and anatomical issues. A clot may also interfere with viewing the larynx, further exacerbating the difficulty of intubation. We describe a pediatric post-tonsillectomy hemorrhage case complicated by a large obstructing clot that was removed with Magill forceps after the airway was successfully secured with an endotracheal tube during rapid sequence induction.

## Introduction

Tonsillectomy is among the most common pediatric surgeries, with over 250,000 surgeries performed annually in children under 15 years in the United States [1]. One of the most common complications is post-tonsillectomy hemorrhage, with a 2.9- 5% incidence [2-4], many of which require a return to the operating room for management. These patients present an anesthetic challenge due to potential anemia, hypovolemia, and increased risk of a difficult airway based on their anatomy. Tonsillectomy is most commonly performed to address obstructive sleep apnea (OSA). Children with craniofacial abnormalities, such as micrognathia or midface hypoplasia, have an increased risk of OSA [5], making intubation more difficult at baseline in many post-tonsillectomy hemorrhage cases. Active bleeding, vomiting, and pharyngeal clots in the setting of a child who may have difficulty cooperating make these intubations particularly challenging. Pharyngeal clots pose a unique challenge to the anesthesiologist since large clots can be difficult to suction. Most published techniques require time that the anesthesiologist cannot afford during a rapid-sequence induction in a child with active bleeding. In this case report, we describe a four-hand technique for safely intubating patients with a large clot obstructing the laryngoscopic view, which can be utilized in situations where the clot cannot be suctioned.

## Case presentation

A 13-year-old female underwent tonsillectomy for OSA and subsequently presented to the emergency department two days after surgery with active bleeding from the right tonsil fossa. Her vital signs on arrival were: temperature 37.1°C, respiratory rate 26, heart rate 104, blood pressure 128/84, and oxygen saturation (SpO_2_) 98% on room air. There were no intraoperative complications, and the patient did not have a history of bleeding disorders. Subjectively, the patient did not feel short of breath despite being tachypneic on presentation. 

During her tonsillectomy, the patient was induced and intubated by the anesthesiologist in the operating room without event. A class 1 view in accordance with the Mallampati classification was observed during intubation. A preoperative airway exam showed adequate thyromental distance and mouth opening, normal temporomandibular joint (TMJ) mobility, intact teeth, and full range of motion of the neck. A Crowe-Davis mouth gag was placed. The soft palate was palpated and showed no evidence of a submucous cleft palate. A rubber catheter was placed through the nasal cavity and brought out through the oral cavity to retract the soft palate. The left tonsil was grasped with an Allis clamp and dissected from its fossa with coblation, and the same procedure was followed for the right tonsil. The nasal cavity was irrigated, the stomach suctioned, and the patient's care returned to the anesthesiologist who extubated without event. She was taken to the postanesthesia care unit (PACU) in good condition.

Two days after her tonsillectomy, the patient presented to the emergency department where she had an episode of large-volume hematemesis and was emergently transported to the operating room for hemostasis after having consented to surgery. IV access was obtained, type and screen were sent, and she received a 750 cc bolus of lactated Ringer’s solution before transfer to the operating room. The patient was anxious on arrival at the emergency department. Her airway exam was unchanged from the prior. The patient was induced and intubated in the supine position. Cricoid pressure was applied and rapid sequence induction was performed with routine doses of fentanyl, propofol, and rocuronium. A MAC 3 blade and a 6.0 cuffed oral Rae endotracheal tube with a stylet were used for intubation by an experienced pediatric anesthesiologist. The direct laryngoscopy view was obstructed by a large pharyngeal clot. Further hyperextension and lifting of the laryngoscope blade allowed for intubation in the hands of an experienced pediatric anesthesiologist on the first attempt. The patient did not desaturate during the management of her airway. Difficult airway equipment was present on standby, including a laryngeal mask airway (LMA), in addition to two Yankauer suction tips. The ENT attending was present in the room at this time.

**Figure 1:**
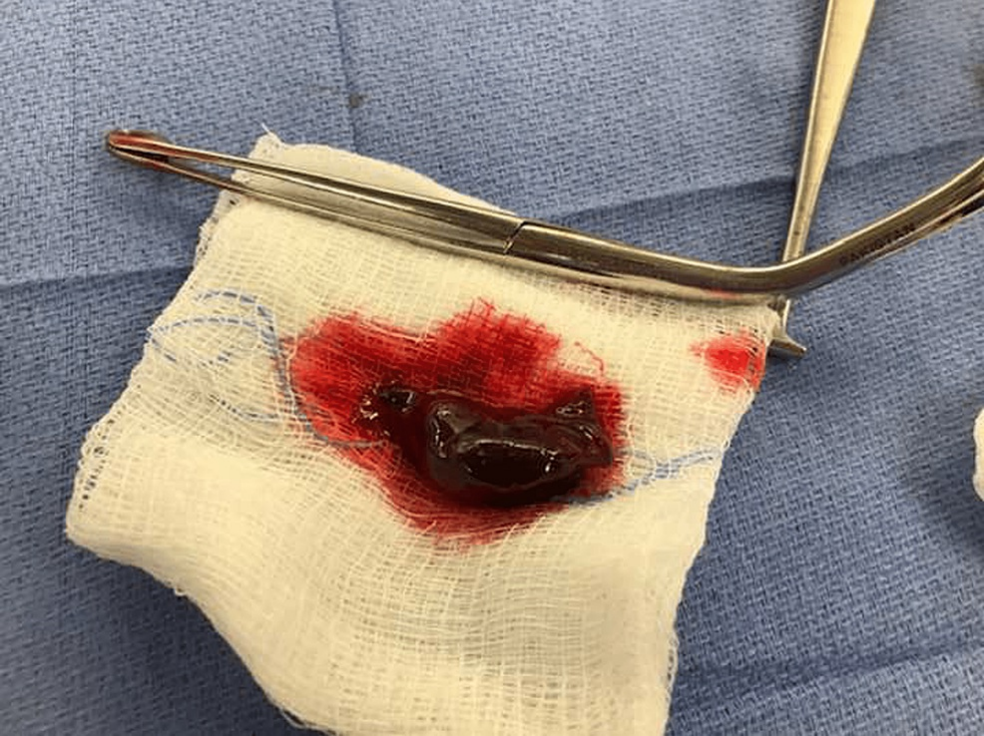
Blood clot visualized after removal with Magill forceps following intubation

**Figure 2:**
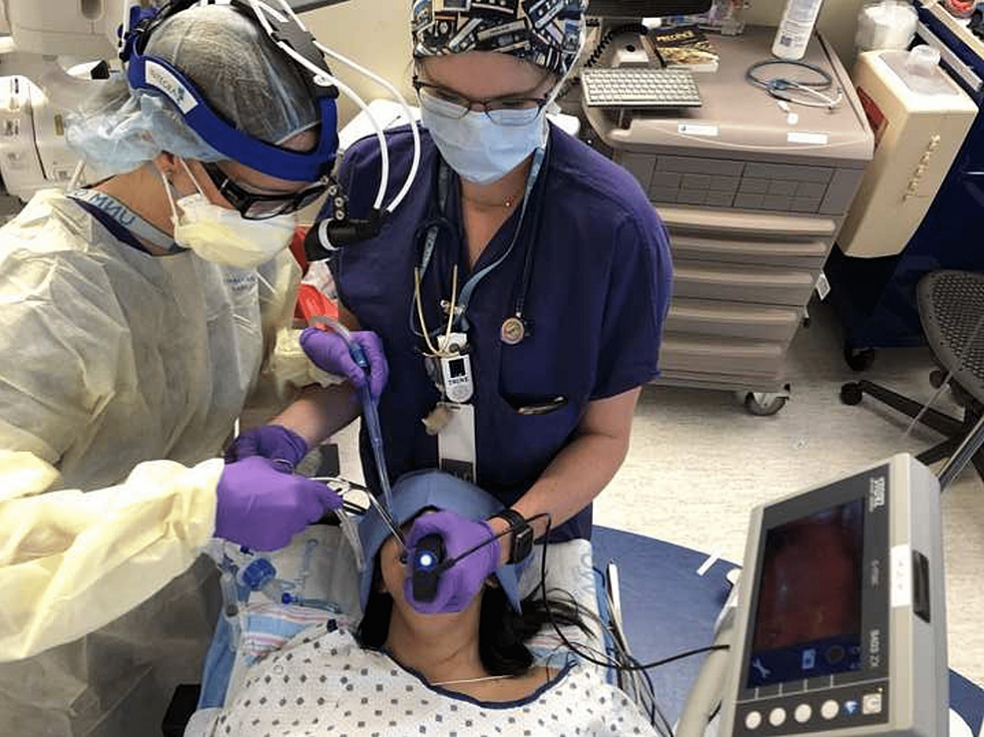
Four-hands technique during rapid-sequence intubation

**Figure 3:**
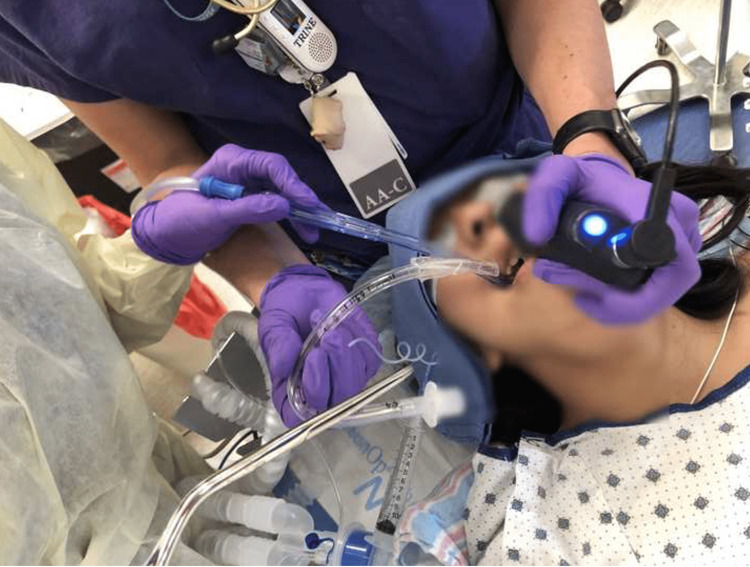
Approach and handling of equipment using the four-hands technique during rapid-sequence intubation

After intubation, the obstructing clot was not removable with full suction using a Yankauer suction tip but was easily removed with Magill forceps (Figure 1). We have these instruments immediately available for this scenario in case intubation is not possible without clot removal. We use a four-hands technique to accomplish this safely. The intubator manipulates the laryngoscope with one hand and inserts the endotracheal tube with the other hand while the assistant holds a Yankauer suction with one hand to address active bleeding and the Magill forceps for possible clot removal with the other hand, using a total of four hands. The assistant optimizes their view using a headlight. This technique provides critical support for establishing a safe airway in a timely fashion (Figures 2, 3).

After intubation and clot removal, the patient was placed in a supine position with a shoulder roll and draped in the usual fashion. A Crowe-Davis mouth gag was placed with a rubber catheter to elevate the palate anteriorly. The tonsil bed was cleared of debris and a large clot on the right was removed with McGill forceps. Intraoperative findings showed a large right tonsillar fossa clot, bright red blood coating the oropharynx, and small arterial active bleeding after clot removal in the right mid-tonsil fossa. The left tonsil fossa had no clot or active bleeding. The bleeding areas were treated with suction and bipolar cautery. The left tonsil fossa was cauterized in areas with granulation tissue. No active bleeding was elicited on that side. The stomach was suctioned and irrigated with copious saline. Clearing the stomach blood required multiple passes, as there were multiple clots. The patient was extubated uneventfully and taken to the PACU in stable condition. She did not experience any emotional distress aside from having anxiety preoperatively. The patient maintained an oxygen saturation of > 92% overnight on room air following surgery without the need for supplemental oxygen. She was discharged the following day following adequate PO intake and pain control. The exam on discharge showed the patient to be alert, comfortable, and in no acute distress. There were no signs of active bleeding from the oropharynx. Respirations were non-labored and there was no stridor. Pulse was regular rate and rhythm. The patient was discharged home in the care of her parents.

## Discussion

A post-tonsillectomy hemorrhage is a known complication of tonsillectomy, however, airway management for patients that require further management in the operating room varies [3-6]. Difficult intubation is not uncommon; one study found that 2.7% of these patients were newly difficult to intubate (e.g. blood in the upper airway, anatomical issues) without intubation difficulty for the initial tonsillectomy surgery [3]. Clots in the airway present additional difficulty; they can interfere with the laryngoscope view and can be difficult to suction, as the clot is held in place within the folds of the pharynx and esophageal inlet. Active arterial bleeding in a patient who is difficult to intubate at baseline presents a further potential risk for intubation and can obscure the camera lens when a video laryngoscope is used.

Multiple techniques are described in the literature for removing airway blood clots, mostly for endobronchial clots [7]. First-line techniques include lavage, suctioning, and forceps extraction (direct or through a flexible bronchoscope), however, these techniques are difficult to perform alone. If unsuccessful, depending on clot location, further options include rigid bronchoscopy, Fogarty catheter dislodgment, and topical thrombolytic agents. These more involved techniques are not practical for patients undergoing rapid sequence intubation. Rapid thrombus removal to allow laryngeal visualization is possible with Magill forceps and suctioning in a four-hands team technique as described. Tonsil forceps or hemostats can be used but do not remove clots as easily since they are not as broad. This prompt coordinated action can be critical in patients with profuse arterial bleeding and/or obstructing clots because clot removal allows better visualization but may also promote further active bleeding. If that occurs, a Yankauer suction tip can be held laterally in the pharynx to prevent active bleeding from further obstructing the view and preventing aspiration. In patients with profuse active bleeding, rapid sequence induction can be initiated with the patient sitting forward over a basin. Once rapid induction is complete, the patient can be placed supine and the four-hands technique can support obtaining an adequate visualization of the larynx for rapid intubation. A video laryngoscope is an excellent tool for many difficult intubations and may be used in this team approach, suctioning to keep the camera view clear can be critical to maintaining a good view. Once the airway is secured, active bleeding can usually be controlled easily with carefully directed gauze pressure using Magill forceps.

## Conclusions

In this particular case, hyperextension of the neck yielded a reasonable view for clot removal and intubation. However, neck hyperextension is unsafe in patients with Trisomy 21 or any other cause of cervical spine instability. Furthermore, patients with a baseline difficult airway from craniofacial anomalies or poor passive range of cervical motion are at higher risk for difficult intubation in the setting of a post-tonsillectomy hemorrhage. When planning an anesthetic in this type of case, it is advisable to anticipate problems such as active bleeding, clot interfering with laryngoscopy, and a newly difficult airway compared to the previous anesthetic. Having the proper equipment, personnel, and ancillary help immediately available until the airway is secured with an endotracheal tube will help maintain control of the airway and reduce the risk of blood loss and aspiration.
